# Comparison of the Results of Hook Plate and Endo-Button Used in the Surgical Treatment of Acromioclavicular Joint Separation

**DOI:** 10.7759/cureus.11987

**Published:** 2020-12-08

**Authors:** Omer Kays Unal, Mirza Zafer Dagtas

**Affiliations:** 1 Department of Orthopedics and Traumatology, Maltepe University, Istanbul, TUR

**Keywords:** shoulder injuries, acromioclavicular joint, orthopedic fixation devices, shoulder functions

## Abstract

Purpose: Our study aimed to compare the clinical outcomes between endo-button and hook plate fixations for the treatment of acute unstable acromioclavicular (AC) joint dislocation.

Materials: A retrospective evaluation of patients with acute AC joint dislocation who were treated between February 2009 and December 2019 was performed. The study was conducted with 39 patients who met the inclusion criteria. Patients were divided into group 1, operated with a hook plate, and group 2, operated with an endo-button. The demographic features and postoperative complications were analyzed. The disability of arm, shoulder, and hand (DASH) scoring system, modified University of California at Los Angeles shoulder score (UCLA) scale, and the visual analog scale (VAS) scores were used to evaluate shoulder functions in these patients. Shoulder functions were evaluated one, three, six, and twelve months after surgery.

Results: There were 21 patients in group 1 and 18 patients in group 2. Gender distribution was 28 male and 11 female, and the average age was 30.9 years (18-50). There were no significant differences in age, sex, side of injury, or follow-up time from injury to surgery between the two groups. The UCLA scores of group 1 and group 2 one month after surgery were 17.2 and 27.2, respectively. DASH scores of group 1 and group 2 one month after surgery were 82 and 52, respectively. The VAS scores of group 1 and group 2 one month after surgery were 70 and 14, respectively. For all scores at first month post-surgery, there were statistically significant differences between groups, but scores became similar 12 months after surgery.

Conclusion: Postoperative shoulder scores of patients with endo-button showed superiority in the early stages. However, after a year of follow-up, the results of the surgery performed with an endo-button or a hook plate were similar.

## Introduction

Acromioclavicular (AC) joint separations are uncommon and occur as a result of both sports and trauma injuries seen in different age groups. It has an overall incidence of 3-4 per 100.000 in the general population [1,2], and it accounts for 3-5% of all shoulder girdle injuries [3].

The AC joint is a movable joint stabilized by several ligaments. AC capsular ligaments provide most of the joint stability in the anteroposterior direction, while the coracoclavicular (CC) ligaments, on the contrary, provide vertical stability [4]. The most common mechanism in AC and CC ligament injuries is a direct force applied to the upper surface of the acromion; It is usually the result of the patient's arm falling in the adducted position [5]. More rarely, AC separation can also occur when the force from falling on an open hand strains the shoulder [6].

In the classification of AC separations, the Rockwood classification in the literature is generally used for both clearly describing the type of dislocation and treatment algorithms [7]. The classification has been made according to the distance of the clavicle to the acromion. After Rockwood defined separation Types I to VI, most authors agree that Type I and II injuries should be treated conservatively and that Type IV to VI are best treated surgically. However, the optimal method of treatment for Type III injuries is controversial [8,9]. Surgery is typically reserved for patients engaged in sports or working activities that are in high demand [10].

In the conservative treatment of AC separation, it is sufficient to restrict shoulder movements for three weeks and then to apply physical therapy procedures [11]. On the other hand, multiple surgical techniques have been employed for AC separation fixation. Using clavicle hook plates, suture buttons, two titanium endo-buttons, excising the distal part of clavicula, and traditional tenodesis methods are examples for these AC separation surgeries [12]. However, the use of hook plates and endo-button techniques in recent years has come to the fore in the surgical treatment of AC separations.

Our study aimed to compare the clinical outcomes between endo-button and hook plate fixations for the treatment of acute unstable AC joint dislocation. The hypotheses tested in this study are that (1) functional shoulder scores of the endo-button group are higher than that of the hooked plate group, (2) there are more complications in the hooked plate group, (3) complaints after surgery are much more common in the patients who were operated with hook plate.

## Materials and methods

A retrospective study of patients with acute AC joint dislocation who were treated between February 2009 and December 2019 was performed. After the approval of the local ethics committee (Maltepe University Ethical Committee of Clinical Researches, Date: 08/08/2020, No: 2020/900/34), the study was initiated, and informed consent was obtained from all study participants. The forty-eight patients that underwent surgical treatment using a hook plate or endo-button fixation systems were enrolled in this study. The inclusion criteria were having acute AC joint dislocation higher than grade III, having isolated AC dislocation, and having available patient records. The exclusion criteria were additional ipsilateral/contralateral clavicle/acromion, humerus fractures or other shoulder pathology, additional rib fractures, chest injuries or other system pathologies, surgery with different surgical techniques other than hook plate or endo-button.

The study was conducted with patients who met the inclusion criteria. The patients were divided into two groups according to the applied surgical technique. Group 1 was composed of patients who were operated with hook-plate, and group 2 was composed of patients who were operated with endo-button. The demographic characteristics and the type of trauma of the patients were recorded. Complications that developed in patients were also recorded.

Surgical technique

Endo-Button Surgery

Operations were carried out under general anesthesia with 1.5 g of cefuroxime axetil antibiotic prophylaxis in the beach-chair position. A 6 cm incision was made to the anterior edge of the distal clavicle from the palpable point of the coracoid. With deep dissection, the coracoid was made visible. The clavicle was reduced, and the clavicle was drilled approximately 3 cm from the AC joint. The drill hole should be directly over the base of the coracoid. It was then drilled all the way throughout the base. When the clavicle was reduced anatomically, the endo-button depth gauge was used to determine the canal length. The endo-button, along with its associated sutures, was pushed into the top of the clavicle through the previously drilled hole and then pushed further into the coracoid drill hole until it reached underside of the coracoid. After the endo-button was placed under the coracoid, the second endo-button was adapted to the upper part of the clavicle, and all threads were tightened. The wound was closed in layers.

Hook-Plate Surgery

Operations were carried out under general anesthesia with 1.5 g of cefuroxime axetil antibiotic prophylaxis in the beach-chair position. The surgical approach was a transverse incision over the lateral third of the clavicle. The acromioclavicular joint was exposed after assessing the torn acromioclavicular ligaments. The fixation of the acromioclavicular separation was done with titanium clavicular hook plate (four, five, or six hole) in templated hook offset (12, 15, or 18 mm) without any supplemental ligamentous repair or reconstruction of coracoclavicular or acromioclavicular ligaments. The wound was closed in layers.

The disability of arm, shoulder, and hand (DASH) scoring system, the modified University of California at Los Angeles shoulder score (UCLA) scale, and the visual analog scale (VAS) scores were used to evaluate shoulder functions in these patients. Shoulder functions were evaluated at the first, third, sixth, and twelfth months after surgery.

Statistical analysis

The statistical analysis was performed using the Statistical Package for Social Sciences (SPSS) version 22.0 (IBM SPSS Statistics, Armonk, NY). The one-sample Kolmogorov-Smirnov test was used to determine the corresponding normal distribution. The descriptive statistics were calculated. Continuous variables with normal distributions are expressed as mean ± standard deviation. To compare group 1 and group 2, the t-test, Mann-Whitney U test, and Friedman test were used for the statistical analysis. Significance levels were set at p < 0.05, p < 0.01, and p < 0.001.

## Results

Thirty-nine patients with closed AC joint dislocations were enrolled in the study. There were 21 patients in group 1 and 18 patients in group 2. Gender distribution was 28 male and 11 female, and the average age was 30.9 years (18-50). Twenty-seven patients had right-side dislocations, and 12 had left-side dislocations. The distribution of the demographic features according to groups is given in Table 1. There were no significant differences in age, sex, side of injury, or follow-up time from injury to surgery between the two groups of patients (p > 0.05).

**Table 1:** Demographic features of the patients N: number, W: women, M: men, R: right, L: left, *Mann-Whitney U

Demographics	Group 1 (n:21)	Group 2 (n:18)	p values*	
Age (years)	30.70 ± 9.65	31.70 ± 8.17	0.065	
Gender (W/M)	6/15	5/13	0.128	
Side (R/L)	14/7	10/8	0.097	
Follow-up (months)	32.4 (26-42)	34.6 (21-45)	0.176	
Complications	8 (36%)	7 (38%)	0.259	

The UCLA, DASH, and VAS scores were assessed to evaluate shoulder function in both groups of patients in the first, third, sixth, and twelfth months after surgery, and the results are summarized in Tables 2-4.

**Table 2:** The mean UCLA scores of the groups SD: standard deviation, *: independent sample t-test

Time	Group 1	Group 2	p values*
1^st ^Month (SD)	17.2 (3.48)	27.2 (7.56)	0.022
3^rd ^Month (SD)	25.4 (3.74)	28.3 (3.46)	0.132
6^th^Month (SD)	28.2 (2.98)	29.9 (3.39)	0.094
12^th^Month (SD)	31.3 (3.14)	30.2 (3.67)	0.086

**Table 3:** The mean DASH scores of the groups *: Mann-Whitney U test

Time	Group 1	Group 2	p values*
1^st ^Month	82	52	0.043
3^rd ^Month	52	44	0.07
6^th^Month	30	30	0.082
12^th^Month	18	16	0.096

**Table 4:** The mean VAS scores of the groups *: Friedman test

Time	Group 1	Group 2	p values*
1^st ^Month	70	14	0.011
3^rd ^Month	18	12	0.065
6^th^Month	16	12	0.162
12^th^Month	12	10	0.091

UCLA score gradually increased postoperatively in patients in group 1, and this improvement became evident after the third month of surgery compared to the first month (p < 0.001 vs. the first month). At the end of twelve months, the mean UCLA score was found to be 31.3 in group 1. Similarly, the UCLA score in patients in group 2 also gradually increased after the surgery. However, this increase had a low acceleration. The mean UCLA score at the first month after surgery was 27.2 and at the end of the twelfth month, it was calculated as 30.2. UCLA score was found to be 31.3 at the end of 12 months in group 1. When UCLA scores were compared between group 1 and group 2, group 2 was statistically superior in the first month after surgery, while the scores of both groups were similar at the end of the 12 months (p < 0.05).

The DASH scores in group 1 decreased gradually postoperatively, with significant improvement starting from the third month after the surgery (p < 0.001 vs. the first month). Similarly, the DASH score in patients in group 2 also gradually decreased after the surgery. The improvement in the DASH scores of group 2 was not statistically significant. The improvement rate of DASH scores in group 1 was higher than group 2 at the end of the postoperative first month. However, at the end of 12 months, DASH scores of group 1 and group 2 were similar.

VAS scores in group 1 decreased significantly at the end of three months. VAS score of group 1 was 70.4 on average at the end of the first month which decreased to 18 at the end of the third month. While the decrease in VAS scores in group 1 continued until the end of 12 months. At the end of the first month in group 2, the VAS mean score was calculated as 14.6, which was very low compared to the hook plate group (p < 00.5). Although the decrease of the VAS score in group 2 continued after the first month. When the two groups were compared in terms of VAS scores, the results were very similar at the end of the twelve months and group 2 showed superiority in terms of the first-month results (p < 0.05). However, there was a significant difference in favor of group 1 in the score change between the first and third months (p<0.05).

Complication rates were similar in both groups. In group 1, three patients had limitation of early motion, two patients had acromion erosion, two patients had arthrosis in the acromioclavicular joint and one patient had an infection. In group 2, three patients had coracoid fractures, three patients had implant failures and one patient had a deep infection. In group 1, the complaints of patients with erosion and early motion restriction resolved after their implants were removed in the third month. All patients with infection in both groups recovered with oral therapy after the bacterial culture and antibiotic susceptibility testing. In group 2, one patient with coracoid fracture was treated conservatively, while two patients with coracoid fractures and three patients with implant failure treated with revision surgery.

## Discussion

In this study, we compared the results of the hook plate and endo-button, which were used in AC joint separations. The results of the study revealed that UCLA, DASH, and VAS scores were higher in the endo-button group one month after surgery, but there was no statistically significant difference between the groups at the end of the 12 months. These results indirectly demonstrate that although the complaints in the early postoperative period after the operation are prominent in patients who have been applied hook plate, there is no difference in long-term results with the endo-button.

AC separation is uncommon and occurs as a result of trauma injuries seen in different age groups. Multiple surgical techniques have been employed for AC separation fixation; however, the use of hook plates and endo-button techniques has come to the fore in the surgical treatment of AC separations in recent years. Arirachakaran et al. reviewed 41 studies reporting results from both methods and reported lower poor function scores and higher postoperative pain in patients treated with the hooked plate [13]. These results were found to be consistent with the first month follow-up results in our study. However, we observed that shoulder functional and pain scores were very close after a year of follow-up. Taleb et al. compared both techniques and revealed that the endo-button technique has superior scores than the hook plate [14]. They examined the functional scores of the patients once and showed the superiority of the endo-button group. However, in our study, these scores were recorded four times postoperatively, and we interpreted the change in scores. As a result, we noticed that the early results of AC separation surgery might change one year after surgery.

Sim et al. reported acromion fractures, plate bending, and AC arthritis as complications in patients undergoing hook plate surgery [15]. Babhulkar et al. reported in their study that acromion erosion started on the 32nd day in the hook plate group [16]. Also, in the literature, acromion erosion and early limitation of motion were the most common complications in patients who underwent acromioclavicular ligament (ACL) repair with hook plates [17,18]. Our study found 36% complications in the hook plate group, which is compatible with the literature, and the most common complications were early motion restriction, acromion erosion, and AC arthritis. In the literature, coracoid fracture and implant failure were the most common complications in construction surgeries performed with endo-button [7,19,20]. We found the most common complications in the endo-button group as implant failure and coracoid fracture. We observed a similar rate of infection in both of our groups, and our infection rates were consistent with the literature [21]. There were no statistically significant differences in the number of complications between groups, but the type of postoperative complication was different between groups.

In the literature, 80% of patients have a loss of reduction, and 20% to 30% of patients undergo re-operation [22]. Subsequently, most of these re-operations are caused by technical errors and a learning curve of the operating surgeon, which must be taken into account when performing AC joint reconstruction [17]. When the literature is reviewed, there are many different revision rates in the hook plate and the endo-button. These studies showing that complications after the hook plate operation are usually treated by removing the plate; on the other hand, endo-button complications were corrected with much more complex procedures [17,23,24]. In our study, revision surgery rates are found similar between groups. Removing only the plate significantly improves the results in the hook group. On the other hand, in the endo-button group, revision surgery is provided with another operation.

The main limitation of our study is that patients were not randomized before undergoing one treatment or the other. The other is that the number of patients is insufficient to obtain more accurate results.

## Conclusions

In conclusion, when we compare the hook plate and endo-button technique in AC separation surgery, the shoulder scores of the endo-button technique show superiority in the early stages. However, after one year of follow-up, the two groups showed the same results. This information suggests that the discomfort of hook plate patients may pass over time.
